# Experiences of mothers of sexually abused children in North-West province, post disclosure

**DOI:** 10.4102/curationis.v39i1.1659

**Published:** 2016-08-16

**Authors:** Gaboipolelwe M. Masilo, Mashudu Davhana-Maselesele

**Affiliations:** 1 Department of Nursing Science, North West University, South Africa

## Abstract

**Background:**

Sexual violence against children is increasing at an alarming rate in South Africa. In 2010 the South African Police Service (SAPS) reported 21 538 rape cases of children under 18 years. In the North-West province (NWP) 5039 incidents of rape cases were reported in 2009. Mothers often experience emotional pain following child sexual abuse disclosure. It is seldom acknowledged that these mothers experience trauma and need support, post disclosure. The researcher has no known evidence of research conducted on the experiences of these mothers in NWP.

**Objective:**

The objective of the study was to explore and describe the experiences of mothers of sexually abused children post disclosure of the abuse.

**Method:**

The research design was qualitative, exploratory, descriptive and contextual. Purposive sampling was used to select mothers of sexually abused children aged 23 to 59 years whose children ranged from 0 to 16 years. Permission to conduct the study was sought from the Provincial Department of Health and informed consent was obtained from the mothers. Interviews were conducted with a sample of *n* = 17 until data saturation. Data were collected through in-depth interviews using a voice recorder and field notes to enhance triangulation. Tesch’s method of open coding was used to analyse data.

**Results:**

Findings indicated that mothers experienced emotional pain post sexual abuse. They expressed shock, anger and guilt for not noticing the abuse. They showed significant depression as a result of lack of support by stakeholders.

**Conclusion:**

Mothers experienced secondary trauma that poses social and psychological challenges with far-reaching implications.

## Introduction

According to Kisanga *et al*. (2012:1) child sexual abuse (CSA) is an international public health problem that has spread to a number of countries needing collective measurers. In a similar vein, Pretorius, Chauke and Morgan (2011:1) acknowledge that mothers of sexually abused children (SAC) experience a significantly high level of psychological distress, and concluded that CSA is a major long-term stressor for parents. According to the United States Department of Justice National Crime Survey (2008:1) there were 248 300 survivors of sexual assault in the USA in 2007, and 15% of these cases were under the age of 12 years, and another 29% were aged 12 to 17. Furthermore, other studies in Asia state that physical sexual abuse ranged from 1.7% in Hong Kong to 11.6% in the Pacific Islands, whilst in Cambodia it ranged from 1.2% to 1.7%, and 1% in Thailand (United Nations Children’s Emergency Fund [UNICEF] 2010:1).

According to UNICEF (2010) the Zimbabwean police reported that CSA continues to increase. The report further indicated that during the week ending 25 September 2012 the cases rose to 81 from 65 the previous week. This indicates the prevalence of this problem.

The South African Police Service (SAPS) report for 2008/2009 indicated that South Africa had the highest number of baby and child rape cases in the world. The 2010 SAPS statistics show that 21 538 rape cases were of children under the age of 18 years. According to the North West province’s (NWP) ‘Integrated provincial strategy to prevent and combat sexual offences’ (2008), reports of sexual assault were high in the North-West province – 137.5/100 000 in 2001/2002 and 130/100 000 in 2006/2007 – and it further indicated that NWP reports an average of 5039 incidences of rape cases annually. These results indicate how pervasive the problem is throughout South Africa. With all these rape cases of children there is a mother who becomes a secondary victim.

After child sexual abuse the mother is confronted by various challenges related to the care and support of such a child including travel to various health facilities to accompany her child, and family disorganisation. Despite the challenges these mothers go through and the emotional and psychological support they give to their children in this process of CSA, few acknowledges the trauma they experience. The researcher observed that these experiences create meta-trauma on an already traumatised mother of SAC post CSA disclosure. This clinical and observational experience provided the impetus for this research on the experiences of mothers SAC in the NWP.

To date, in NWP there is little disseminated research available which has explored and described the experiences of mothers whose children were sexually abused.

### Problem statement

According to Tavkar (2010:1) CSA continues to be prevalent globally and the researchers and practice focus on the pain experienced by SAC, whilst their mothers are largely overlooked. The researcher also found that there was a dearth of literature regarding the experiences of mothers of SAC post disclosure in NWP and that researchers on experiences of child sexual abuse focus on the child, but not the mother who often experiences secondary trauma.

The researcher then explored and described the experiences of mothers of SAC post disclosure in NWP. It was against this background that the following research question arose: What are the experiences of mothers of SAC post sexual abuse disclosure?

#### Purpose

The purpose of this study was to explore and describe the experiences of mothers of SAC post disclosure, with the aim of developing recommendations.

#### Significance of the study

The research findings of this article could assist in developing recommendations to support the mothers of SAC. The health care providers could inform curriculum developers on health care training for professionals to include introduction of forensic nursing and counselling skills in undergraduate programmes to create a climate that would support mothers of SAC. The findings would assist in addressing the plight of mothers in areas such as self-blame and destructive behaviour. It could also contribute to the practice because legal system and communities will see the need to support the mothers. Researchers in the NWP could investigate the other strategies that could be put in place to assist mothers of SAC to cope with the secondary trauma.

### Definition of concepts

**Child**: This refers to either a male or female of any race under the age of 16 years who was sexually abused within 12 months prior to the commencement of data collection.

**Child sexual abuse:** This is forced sexual intercourse with a child who is 16 years old and younger.

**Mother of a sexually abused child**: This means any non-offending woman, stepmother, caregiver, foster parent, adoptive parent, grandparent or biological mother who takes care of the SAC up to 16 years.

**Rape**: Rape is the forceful sexual penetration committed by a man with intention of having sex for gratification or other coercive and punitive cause with a child up to 16 years.

**Post sexually abuse disclosure:** This means the period after child sexual abuse activity is revealed.

## Research design and methods

An exploratory, descriptive and contextual qualitative design was employed with the aim of exploring and describing the experiences of mothers of SAC post disclosure. The data gathered from mothers of SAC were also described so that the researcher could reach an in-depth understanding of the experiences and therefore clarify to a wider audience the impact of this coercive act. The research design was contextual because it purposively identified mothers of SAC as principal informants.

### 

#### The study population and sample

The population comprised mothers whose children had experienced sexual abuse; their names were identified in the sexual assault register at the care centres in the NWP.

The sampling criteria were that the mother was a primary caretaker of any SAC between 0 and 16 years, sexual abuse must have occurred within one year prior to the data collection.. The sexual abuse included oral, vaginal or anal penetration from a male adult perpetrator; this included incest. The SAC was either a male or a female and the CSA must have occurred in NWP. Mothers were excluded from the sample if they were accused of physical or sexual abuse, and those who were not mentally sound.

#### Data collection

The researcher used individual unstructured in-depth interviews, field notes and a voice recorder to collect data. The selected participants were informed about the problem under investigation so that they could provide relevant information (Sarantakos 2013). The in-depth interview was deemed appropriate to determine the participants’ experiences, opinions and their reactions towards the disclosure of their children’s sexual abuse (Brink, Van der Walt, & Van Rensburg 2012:158). The aim was to understand the participants’ responses to the research question in the wider context of the interview (De *Vos et al*. 2011). The researcher used the following question to facilitate the interview: ‘What are your experiences following your child’s sexual abuse disclosure?’

The researcher had follow-up questions depending on the participant’s response to the principal question and the subsequent questions probed participants for detailed amplification that facilitated exploration of the experiences relating to secondary victimisation. Each interview took 45 to 60 minutes to complete.

#### Data analysis

Data were captured, audio-recorded, translated and transcribed from Setswana into English. Codes and coding were developed for the analysis. Themes were categorised using symbols. This analysis was carried out using Tesch’s eight steps (Creswell 2014). The raw data were also sent to an independent coder for comparative analysis. The coder and the researcher met to interrogate and tease the themes that emerged.

### Ethical considerations

The researcher obtained ethical clearance from the North-West University research ethics committees whilst permission to conduct the study was obtained from the North-West Provincial Department of Health. Written informed consent was obtained from the selected participants and they were made aware that participation was voluntary. In the consent form the rights of the participants were clearly spelt out. They were informed that they had the right to withdraw or to refuse to participate in the study. Participants were assured of confidentiality, anonymity and privacy.

## Trustworthiness

To ensure trustworthiness the criteria of credibility, dependability, applicability and conformability were used Lincoln and Guba (1985:290) and Polit and Beck (2010). Credibility was ensured by identifying participants whose children had experienced sexual abuse. The researcher had prolonged engagement in the field with the participants to gain their trust (Brink *et al*. 2012). The researcher checked with peers to ensure accuracy of information given. Recordings were replayed for the supervisor to ensure credibility (Polit & Beck 2010). The researcher had the supporting documents reviewed by the second reviewer as suggested by Skhosana and Pen (2009).

The researcher provided sufficient descriptive data in the research findings so that it can be evaluated for applicability to other settings (Polit & Beck 2010). The purposive sampling was used to confirm that the sample selected bears the characteristics that were premised in the research question under study. This study was consistent across the whole research process, guided by the research questions and objectives (Brink *et al*. 2012; De Vos *et al*. 2011).

An independent coder was given the tape to confirm the interviews by listening to the recordings of the interviews. The researcher set aside preconceived ideas about the consequences of trauma on mothers of SAC (Brink *et al*. 2012).

## Results

Three broad themes, categories and sub-categories emerged from the data collected and the results from all participants are summarised in and discussed following Table 1.

**TABLE 1:** Summary of findings.

Theme	Category	Sub-category
Reaction of participant to disclosure of child sexual abuse	Emotional and Psychological reaction	Guilt feelings and self-blame upon discovering CSA. Experience of pain post disclosure. Depression as a result of disclosure of CSA. Suicidal ideation.
	Socio-economic reaction	Self-isolation to protect the child. Financial constraints.
	Spiritual reactions	Coping through religious beliefs. Coping through belief in supernatural powers.
Effects of child abuse on a child as viewed by mothers	Physical and psychological trauma observation	Physical trauma observed by mothers. hild experience of psychological trauma.
	Feelings for revenge on perpetrators	Revenge on perpetrators.
Experiences regarding support	Experiences regarding medical support	Experiences regarding interaction with the multidisciplinary team that attended to their children.
	Experiences related to legal support	Views about support received from police and judiciary.
	Experiences related to societal support	Views about support from family. Neighbours’ willingness to support. Community willingness to support. The need to form support groups.

*Source*: Authors’ own work

### Reaction of the participants to disclosure of child sexual abuse

Mothers of SAC suffer secondary trauma immediately when they became aware of CSA disclosure. From this the following categories emerged: emotional and psychological, socio-economic, and spiritual reactions.

### Emotional and psychological reactions

Discussions with participants revealed that mothers of CSA suffered emotional and psychological trauma. This is discussed under the following sub-categories: guilt feelings and self-blame after discovering CSA, emotional and psychological pain post disclosure, depression as a result of disclosure of CSA and suicidal ideation.

### Guilt feelings and self-blame after discovering CSA

Participants expressed experiencing guilt feelings and self-blame upon discovery that their children had been sexually abused.

One participant explained her experiences as follows:

‘The perpetrator is my sister’s son, he is 22 years old. I felt pity for him, he was not employed. I accommodated him so that I can help him to look for work. I really feel guilty to have done that. He got work, now he has sexually abused my 18-month old baby.’ (P1)

This participant cried during the interview. There was an overwhelming sense of self-blame; she felt she had been part of the whole process that led to the abuse of her child. Myrick and Green (2013:196) make similar observations that, where the perpetrator is a family member, mothers feel betrayed and unable to trust their own relationship. The participant’s response demonstrates a mother’s relationship to the perpetrator which is linked to her emotional and psychological responses to the incident.

This participant reflected as follows: ‘I feel like I am irresponsible. I am angry with myself. I wish what happened to my baby could have happened to me, but not to an innocent soul.’ (P5)

This reflection is supported by Smith *et al*. (2010:264) who demonstrate that parents of SAC may experience feelings of guilt, sadness, worthlessness, uncertainty and that they have immense difficulties in channelling their anger.

### Experience of emotional and psychological pain post-disclosure

Most participants endured emotional pain following their children’s sexual abuse and it was not easy to forget what they had experienced. One participant expressed her emotional pain as follows:

‘What makes me very sad is that the child is afraid and scared. At night she would take the couch and close the sliding door with it, even if the door is closed. She’s even afraid to go to the toilet inside the house all by herself. This is an in explainable and unforgettable pain in me.’ (P12)

Carvalho, Galvao and Cardoso (2009:500) concur that mothers reveal inexplicable and deep pain as evidenced by their difficulties in overcoming the experience of seeing their daughters who have been victimised.

Yet another participant expressed her emotional pain in the following words:

‘My twins, 16 years old, confessed that they have been sexually abused by the same person … my uncle’s son, a traditional healer. I decided to lay charges against him and it became painful because while the case was on, my twins were still meeting him … and one of them dropped the case. This is painful because my father also says I should not have gone to police because he is my cousin.’ (P7)

It is apparent that social orientation and traditional norms around gender roles are the major contributors to CSA in South Africa. In this latter case the close relative is also a healer, which is a venerable position in the society. Because of the esteem associated with such a position, reporting the incident to the police by the relative who actually warn against it, is perceived negatively by the rest of family.

### Depression as a result of disclosure of CSA

One response from a participant captures this anxiety: ‘I felt like my vessels on the head were engorged; I had difficulty putting my head on the pillow. Imagine a 14 year old pregnant girl after being raped by six boys.’ (P17)

In line with the above participant’s experiences of depression, Tavkar and Hansen (2011:195) indicated that upon hearing that a child has been sexually abused, the non-offending mother may feel immobilised, overwhelmed, experience severe emotional trauma and may also develop a variety of symptoms, such as clinical depression, loss of trust in the safety of others, anxiety and severe guilt for not noticing that her child has been sexually abused. In this study, the participant’s narrative version supplies evidence that gang rape activity is an aberration globally and in South Africa, and a matter of serious concern.

### Suicidal ideation

One other participant recounted the sexual abuse of her 16 year old pregnant daughter in the following words:

‘I nearly killed myself. When I heard it, she was already at care centre. She phoned me and said “Mom I am raped, my panty is on the doorway you will see it. I feel like killing myself.”’ (P8)

This participant’s child was raped whilst she was pregnant. She was raped by her cousin and his friend. The intention to kill herself is evidence of suicidal ideation.

In line with the above expressions of the participant, McDonald and Tijerino (2013) spoke of depression, isolation, self-harm and other negative effects of CSA; all of these effects are common to survivors of sexual violence

### Socio-economic reaction

The category on socio-economic reaction is discussed under the following sub categories: the need for self-isolation to protect the child as well as financial constraints.

### Self-isolation to protect the child

A few mothers indicated that they isolated themselves from their family, friends and community because they did not want to be blamed, stigmatised or ostracised for something that they could not be personally held to account for. Some of the reactions observed were behavioural such as being withdrawn and a mother isolating herself.

A participant said this with bitterness:

‘I was scared, he could have killed him; it is only by luck that he is still alive. He could have killed and hide him somewhere. Those are the things that make me isolate myself from the rest of the society. How can I live in such conditions of being seen as an irresponsible parent who cannot protect an innocent soul … a mother who is not looking after her child? People are not friendly anymore.’ (P10)

The observation of the participant is supported by (Tavkar 2010:13) who stated that mothers of SACs suffer psychologically; this includes social isolation, and displaced anger towards the family and the perpetrator.

In this article, mothers isolated themselves with the aim of protecting the child from abusive environments and from being treated as outcasts. Mothers were critically affected by different environments such as the family, community influences, as well as cultural and societal attitudes.

### Financial constraints

As the majority of the interviewees are unemployed, participants expressed lack of finance as a barrier to travelling to institutions where the child is medically required to go, to obtain an identity document, as well as to buy food for the family. Most of them indicated that they were not receiving any social support.

A mother who did not have transport money to take the child for an appointment at care centre said:

‘Yes! I was hurt because those people caused me many problems as I’m unemployed, my children do not receive any grants. My children and I cannot receive social grants because I do not have ID, so I am running after piece jobs so that I can have money to buy food. If I am to take her to the clinic where will I get the money from? They even deprived me of my job and they messed up with my children’s food. This situation gives rise to more financial problems.’ (P4)

This quote strongly suggests that poor socio-economic conditions contribute to mothers not honouring appointments given by professionals at care centres. Richter and Dawes (2008:86) reported that the high levels of unemployment in South Africa result in family stress, which lead to punitive behaviour towards children. This is supported by Mork, Sjogren and Svalerd (2013:367) who stated that unemployment and underemployment are significant household stressors that cause maladaptive behaviour to deal with CSA in the family. This article found that there is lack of supervision because many parents leave their children alone or with relatives because of a lack of places of care.

### Spiritual reactions

In this category participants’ different experiences are discussed under the following sub-categories; coping through religious beliefs, and coping through beliefs in supernatural powers.

### Coping through religious beliefs

Given the challenges associated with child rape, many mothers resort to some escapist solution reflected as follows: ‘I relied on reading the Bible that is where I received comfort. After scripture reading I felt relieved.’ (P2)

Willingham (2007:16) confirmed similar experiences by reporting that mothers had mixed feelings and inner conflicts because of their cultural and religious beliefs. Contrary to Meichenbaum (2008:7), Jones and Trotman Jemmott (2009:94) found in their study that religion is an important aspect of life to the majority of respondents and that this is a general tendency in South Africa. It is apparent that some mothers relied on some spiritual intercession in order to cope post CSA disclosure whilst others rely on culture.

### Coping through beliefs in supernatural powers

Some participants in this study indicated that they were directed by ancestral supernatural powers to take particular actions.

One participant said: ‘I don’t know why this boy is doing this, raping his sister. Are the ancestors punishing me ot is it witchcraft?’ (P9)

There is dearth in literature that supports the perception that CSA perpetrators do it because of witchcraft or ancestors who can determine the actions to be taken by guardians. Many of the mothers who were interviewed believed that their ancestors were punishing them whilst others thought that incest is a consequence of witchcraft.

### Effects of sexual abuse on a child as viewed by mothers

Two categories emerged from this theme; physical and psychological trauma observations, and the desire for revenge on perpetrators.

### Physical and psychological trauma observations

Many responses suggest levels of physical and psychological trauma observation:

‘The doctor says the vagina and the urinary bladder are torn, and I don’t know where the faeces come from. I am told that an operation is very expensive. [*Crying, covering head with dress, standing up and sitting down, very restless* *… tears rolling down her cheek*] Her genital is torn, this is a life-time trauma on me … It is unbelievable, my 10 year old child. The doctor says she needs reconstructive surgery.’ (P3)

The physical trauma on the victim is impossible to measure.. This affects the mother who insists on the physicality of the trauma and the implications thereof on the future of her child. Another participant articulated this experience in plain terms: ‘The private parts were red, swollen and bruised. The baby did not want me to touch her to remove pampers.’ (P1)

In support of these quotes, Allnock and Start (2010:2) and Kisanga *et al*. (2012:16) found that mothers of SAC expressed that their children experienced physical trauma at an unacceptable age. The physical trauma experienced by children left mothers in horror and with disbelief, often with symptoms of post-traumatic stress disorder.

### Child experiences of psychological trauma

A participant whose five year old son was sodomised said after the disclosure:

‘I can’t say there aren’t any changes because he was doing fine at school then after the incident he became forgetful, right now he failed his grade. He was not like this. He is no longer performing well at school.’ (P13)

She foreshadows this narrative in traumatic terms; the depressed performance of the sodomised boy, forgetfulness and the massive change is captured in the words, ‘He was not like this’.

Another participant echoed similar experiences: ‘Yes she has changed, she is trying to make herself strong, but she is no longer like before. It seems like she experiences flashbacks and she gets scared.’ (P12)

These statements about psychological changes that mirror represented feelings and anxiety are supported by the study conducted by McDonald and Tijerino (2013:9) that revealed a subtle difference that existed in the responses to CSA and also found that both genders show equally adverse mental health outcomes. In this article, mothers observed that their children were left with frightening memories, constant feelings of being endangered and that they were unable to trust people, especially men. The mothers also experienced that CSA affects children’s education: their attention span, behaviour and mental status showed that they could not concentrate in class, as evidenced by the failure to perform academically at school. Children felt vulnerable, helpless, unsafe, and they experienced trauma, and were anxious and tend to withdraw from other children. They also experienced flashbacks of the rape incidents.

### Need to take revenge on perpetrators

The mothers felt that the perpetrators did not deserve to live. One participant suggested: ‘The solution is to kill this man, he is a dog. I want him dead. Imagine almost every day taking the child to an RDP house to abuse her sexually.’ (P3)

Such intense loathing of the perpetrator comes from deep-seated emotions. Jones and Trotman Jemmott (2009:16) concur with the mothers’ utterances, adding that perpetrators should be severely punished and that law enforcement is ineffective because perpetrators of CSA are not apprehended, nor given punitive custodial sentences if they are. It is concluded that this horrific action requires a more comprehensive, systematic approach to identify the best legal practices to apprehend the perpetrators.

### Experiences regarding support participants received, both formally and informally

From this theme, three categories were identified, namely, experiences related to medical, legal and societal support.

### Experiences related to medical support

From this, the following sub-category emerged; experiences related to the interaction with the multidisciplinary team that attended to their children.

### Experiences relating to interaction with the multidisciplinary team that attended to their children

Participants expressed the wish that they wanted their children to be seen and examined by a multidisciplinary team, but in most cases the team was composed differently. Mothers reported that some professionals were good whilst others did not show much interest in examining the child.

A participant said, ‘When we explained our problem to them, we never stood in the queue. The person who was testing her was heartbroken that she was raped sleeping in the house.’ (P11)

Another participant echoed this after being in a queue for a long time:

‘My child was not the only one. The parents of children who experienced this received letters from the school and appointments at centre were made according to dates. We went to the care centre but waited – it took long, it seemed as if no one cared. They knew that we were coming, but when we arrived there it was as if no one knew. The time was 16:00 when we were helped.’ (P14)

Smith *et al*. (2010:263) indicate that participants who reported unprofessional treatment by nurses or not receiving information concerning the status of physical examination may be victimised and therefore not likely to be engaged in help-seeking behaviour. Smith is supported by Kim, Trickett and Putnam (2010:621) stating that professionals who work with mothers of SAC may need to assess the current psychosocial functioning and childhood parenting practices. Such interventions for the mothers of SAC may increase the possibility for resilience in the SAC. Mothers expressed discordant interpersonal relationship experiences with multidisciplinary teams. Some reported having received good care from the multidisciplinary team whilst others complained about the negative attitudes of health professionals who demonstrated no empathy.

### Experiences related to legal support

Participants expressed different views regarding the support they received from the legal fraternity.

One participant talked about the unprofessional behaviour displayed by those who were meant to arrest the abuser in the following narrative:

‘He is arrested, but I have lost confidence in our judiciary, even police, they will release him saying there is no evidence. They did that with another daughter of mine. The pain is aggravated by the fact that the police officer who is handling this case has never shown up, he keeps on sending SMSs saying he will come, look at this SMS [*showing the researcher*]. It is two months now, but nothing is happening.’ (P6)

Adefolalu (2014:346) concurs that in some cases the perpetrators are not reported to the police because only a few cases reach trial. Contrary to the above, this research provides evidence that some police officers were supportive to mothers of SAC. It is evident that some police officers do not see CSA as a serious crime because they do not arrest the perpetrators. Police officers should be aware that they increase maternal trauma if they do not assist these mothers post disclosure.

### Experiences related to societal support

The mothers of SAC experiences are discussed in the following four categories.

### Views about support received from the family

One of the participants said, ‘The day I came here, I was with my aunt. My family is supporting me, my husband, sisters they phone nearly after every two days.’ (P12)

Smith *et al*. (2010:266) agree with the participants’ submissions that a family systems’ approach, supplemented by a Western perspective, should be adapted to address the needs of South African CSA survivors. Some participants received support from their family members and seemed to cope with the reality of SAC.

A participant expressed lack of support by some family as a major concern in this manner:

‘My mother stayed with us for a while, she complained that I should have called her first before I lay charges for my son who sexually abused his 5-year old sister. My mother was totally not supportive … she blamed me.’ (P11)

Myrick and Green (2013:195) supported the participants’ reflections by reporting that the study conducted found that the parents and families experienced significant emotional stress following a child’s disclosure of sexual abuse. Parents and family members of SAC may experience feelings of worthlessness (Smith *et al*. 2010). In this study other family members did not agree with the idea of laying charges against the perpetrator, especially if it is incest.

### Neighbours’ willingness to support

Some neighbours to mothers of SAC were willing to assist where necessary.

One participant said, ‘One of my neighbours gave me her cell phone numbers and said I should call him when I want to go to the clinic, and she keeps on calling me to hear about how the case is going.’ (P13)

Pretorius *et al*. (2011:8) confirm that the society, community, neighbours and friends often blame mothers of sexually abused children for allowing sexual abuse of their children to happen. These researchers’ results are contrary to the findings of this article where neighbours were supportive. Contrary to other CSA studies, in this article neighbours were supportive and assisted victims.

### Community members’ willingness to support

Most participants during interviews reported receiving positive support from community members. One of the participants said: ‘Teachers observed that the child is withdrawn, does not write, talk, always crying and poor school progress.’ (13)

Bein (2011:13) supported the idea that the community must support the mother of SAC by advocating that institutions such as churches and schools should support the survivors by engaging in some activities. In this article the community had been supportive.

### The need to form support groups

Mothers indicated that they needed support of other mothers who had experienced the same ordeal. One other participant said:

‘I want to meet people like me who experienced the same trauma so that we can hold some group discussion so that one can get an idea of how others coped. The parents must also receive counselling.’ (P13)

Myrick and Green (2013:199) argue that non-offending mothers, after disclosure of CSA need support from therapists and other professionals throughout the process of investigation and also immediately after disclosure. They further indicate that more valuable and social support may include support groups, treatment providers and survivors’ advocates. Mothers clearly need support from all stakeholders so that they, in turn, can support their children during their traumatic stage, as they themselves are not recognised as secondary victims of CSA.

## Conclusion

This article revealed that participants blame themselves for the CSA; they feel that they were betrayed by family members. The article further suggests that mothers should be comprehensively supported post CSA disclosure so as to prevent self-blame and guilt that may lead to poor coping strategies, depression and post-traumatic stress disorder (PTSD).

Many mothers demonstrated psychological and emotional symptoms. The researcher observed that mothers exhibited high symptoms of depression and PTSD. Participants were observed to be pre-occupied by what had happened to their children. They were distressed because they were faced with multi-tasks of protecting the children, from being re-victimised by the perpetrator and supporting the child during the healing process and to deal with their own psychological and emotional stress induced by CSA disclosure and the indifferent attitude of the professionals.

The distress experienced by mothers of SAC is related to their lack of social support and inadequate coping strategies they employ to deal with their trauma. Their indication of a desire to commit suicide is to avoid experiencing future pain and suffering of their children from CSA. Mothers also entertained the killing of perpetrators as a solution, justified by the fact that perpetrators had inflicted unforgettable pain on their children.

Mothers observed that their children were left with frightening memories, a sense of constant endangerment and that they were unable to trust people, especially men. The mothers displayed high symptoms of depression and reduced self-esteem and self-worth. They were tearful and reported not to being able to cope with the emotional trauma. They isolated themselves with the aim of protecting the child from the abusive environment and from being treated as an outcast. Participants reported feelings like hiding from the public after the CSA disclosure. This article identified that most participants live in poverty, mainly because of unemployment; as a result they do not take their children to the health services for follow-up visits. They leave their children alone to search for temporary jobs and this puts the children at risk of being sexually abused. The article found that the impact of CSA disclosure and financial constraints rendered the mother ineffective to take preventive decisions.

It revealed that religion and spirituality are linked to the mothers’ identities as human beings. Mothers in this study questioned God about their children’s experiences. It was also found that stress can decrease if prayer is used as a form of intercession to God. Mothers did not take their children to care centres or failed to lay charges against perpetrators in the hope that God would intervene on their behalf. They said they believed that God would always be on their side. They claimed to be hearing voices that gave them direction about how to handle the perpetrators. Some of them concluded that perpetrators are bewitched.

Mothers need information from various stakeholders so that they are aware of the differences between supernatural powers and the real power of God. Some participants claimed that God and ancestors helped them to take decisions.

The pain expressed is a fundamental characteristic of the emotive life which evidences the certainty of permanent trauma. It is difficult to overcome it because it remains after disclosure and destroys a person’s emotional, physical, psychological, spiritual and socioeconomic well-being.

The findings of this study are supported by Kisanga *et al*. (2012:16) who indicated that rapists were known to survivors; the majority of perpetrators were family members, neighbours. Mothers felt an urgent need for revenge, they suffered anguish, and expressed anger and rage towards the perpetrators and their family members. Perpetrators ought to be prosecuted, not to be seen on the street, yet they are granted bail by the justice system; there is general leniency towards sexual offenders.

This article substantiated the need to support mothers of SAC. If an appropriate intervention is not employed, families may continue in a dysfunctional environment. Findings revealed that mothers sought professional counselling services after disclosure of CSA. Counselling should include individuals, groups and family members. Professionals should provide sessions of psychotherapy and counselling to debrief the mother so that healing and closure could be facilitated for the mother. It is imperative to find strength to give the mother first level emotional support and debriefing after CSA disclosure.

## Limitation of the study

The study only focused on mothers of sexually abused children post disclosure; yet, during interviews it was discovered that some fathers had accompanied their children to care centres. The study was conducted only in NWP, therefore it cannot be generalised to other provinces.

## Recommendations

The recommendations of this article regarding research, clinical practice, education and policy making are discussed in the subsections that follow.

### Research

More research is needed in the NWP to investigate the other strategies that could be put in place to assist mothers of SAC to cope with the secondary trauma.

### Clinical practice

The care centres in the province should collaborate with other institutions to develop a standardised and integrated multidisciplinary approach to support mothers of SAC.

### Education

Introduction of a forensic module in undergraduate programmes, such as support of non-offending mothers of SAC following disclosure, would be a welcome addition to broaden the curriculum.

### Policy makers

Policies to manage mothers of SAC post disclosure must be included in the primary health care package.
